# Prevalence Rates of Arterial Hypertension According to the Threshold Criteria of 140/90 or 130/80 mmHg and Associated Cardiometabolic and Renal Factors: SIMETAP-HTN Study

**DOI:** 10.3390/medicina59101846

**Published:** 2023-10-17

**Authors:** Vicente Pallarés-Carratalá, Antonio Ruiz-García, Adalberto Serrano-Cumplido, Ezequiel Arranz-Martínez, Juan Antonio Divisón-Garrote, Ana Moyá-Amengual, Carlos Escobar-Cervantes, Vivencio Barrios

**Affiliations:** 1 Health Surveillance Unit, Mutual Insurance Union, 12004 Castellon, Spain; 2 Department of Medicine, Jaume I University, 12006 Castellon, Spain; 3 Lipids and Cardiovascular Prevention Unit, Pinto University Health Centre, Pinto, 28320 Madrid, Spain; 4 Department of Medicine, European University of Madrid, Villaviciosa de Odon, 28670 Madrid, Spain; 5 Family and Community Medicine, Getxo, 48993 Bizkaia, Spain; adal1953@hotmail.com; 6 San Blas Health Centre, Parla, 28981 Madrid, Spain; ezequielarranz@gmail.com; 7 Casas Ibáñez Health Center, 02200 Albacete, Spain; jadivison@telefonica.net; 8 Santa Catalina Health Centre, 07001 Palma, Spain; anamoya48@gmail.com; 9 La Paz University Hospital, 28046 Madrid, Spain; escobar_cervantes_carlos@hotmail.com; 10 Ramon y Cajal University Hospital, 28034 Madrid, Spain; vivenciobarrios@gmail.com; 11 Department of Medicine, Alcala University, 28801 Madrid, Spain

**Keywords:** arterial hypertension, blood pressure control, cardiovascular diseases, cardiovascular risk factors, prevalence

## Abstract

*Background and objectives*: Arterial hypertension (HTN) is the leading preventable cause of atherosclerotic cardiovascular diseases (ASCVD) and death from all causes. This study aimed to determine the prevalence rates of HTN diagnosed according to the threshold diagnostic criteria 130/80 mmHg and 140/90 mmHg, to compare blood pressure (BP) control, and to evaluate their associations with cardiovascular diseases and cardiometabolic and renal risk factors. *Materials and Methods*: This was a cross-sectional observational study conducted in primary care with a population-based random sample: 6588 people aged 18.0–102.8 years. Crude and adjusted prevalence rates of HTN were calculated. BP control was compared in HTN patients with and without ASCVD or chronic kidney disease (CKD). Their associations with cardiovascular diseases and cardiometabolic and renal factors were assessed using bivariate and multivariate analysis. *Results*: Adjusted prevalence rates of HTN diagnosed according to 140/90 and 130/90 criteria were 30.9% (32.9% male; 29.7% female) and 54.9% (63.2% male; 49.3% female), respectively. BP < 130/80 mmHg was achieved in 60.5% of HTN patients without ASCVD or CKD according to 140/90 criterion, and 65.5% according to 130/80 criterion. This BP-control was achieved in 70% of HTN patients with ASCVD and 71% with CKD, according to both criteria. Coronary heart disease (CHD), heart failure, atrial fibrillation, stroke, diabetes, prediabetes, low glomerular filtration rate (eGFR), hyperuricemia, hypercholesterolemia, obesity, overweight, and increased waist-to-height ratio were independently associated with HTN according to both criteria. *Conclusions*: Almost a third of the adult population has HTN according to the 140/90 criterion, and more than half according to the 130/90 criterion, with a higher prevalence in men. The main clinical conditions associated with HTN were heart failure, diabetes, CHD, low eGFR, and obesity.

## 1. Introduction

Arterial hypertension (HTN) constitutes a major global public health problem according to the World Health Organization (WHO) [1] and particularly in primary care, the reference setting for screening, diagnosis, initiation of treatment, and follow-up of most of patients with HTN. Currently, it continues to be the single most important modifiable risk factor, being responsible for the high burden of cardiovascular morbidity and mortality and all-cause mortality [2,3], in addition to being the main risk factor for the development of chronic kidney disease (CKD) and disability-adjusted life years [2,4].

HTN causes 10.4 million deaths annually worldwide [3,5,6]. In 2010, it was estimated that 1390 million people in the world had HTN [7], with an increasing trend, especially in high-income countries [8,9]. In Europe, HTN affects more than 150 million people, with a prevalence of 30% to 45% in adults and age-adjusted prevalence rates of 24.1% in men and 20.1% in women, being over 60% in people older than 60 years [5,9]. In 2019, the global age-standardised prevalence of HTN in adults aged 30–79 years was 33% [10]. According to the National Health and Nutrition Examination Survey (NHANES III) conducted in the United States between the years 1988 and 1994, the HTN age-adjusted mortality was 14.3/1000 person-years, and more than 50% of mortality was caused by coronary heart disease (CHD) and stroke [11]. The Spanish IBERICAN study showed that the HTN prevalence in patients attended in primary care was 48.0% [12]. The direct and indirect costs attributable to HTN ranged between 5.6% and 7.5% of Spanish health spending [13], with both the degree of knowledge (around 60%) and overall control (around 25%) remaining very low [14].

Epidemiological studies show that cardiovascular risk (CVR), renal morbidity or fatal events start from an office systolic blood pressure (SBP) > 115 mmHg and diastolic blood pressure (DBP) > 75 mmHg [15]. The office threshold blood pressure (BP) values correspond to the BP level at which the therapeutic benefits with treatment exceed those of inaction [16]. The European Society of Cardiology (ESC), European Society of Hypertension (ESH), European Renal Association (ERA), and International Society of Hypertension (ISH) consider that the threshold levels of SBP and/or DBP to define HTN are ≥ 140/90 mmHg [16]. On the other hand, the American College of Cardiology and American Heart Association (ACC/AHA) consider that threshold SBP/DBP values to define HTN are ≥ 130/80 mmHg [17]. However, the BP control targets (<130/80 mmHg) in HTN patients are similar in both guidelines [18].

The WHO proposes achieving the global goal of reducing the HTN prevalence rate by 25% in 2025 (https://www.who.int/es/news-room/fact-sheets/detail/hypertension [accessed on 31 August 2023]). Studies about the prevalence of cardiovascular risk factors (CVRFs) are necessary to improve cardiovascular prevention activities, to adequately plan the necessary health resources, and to monitor and evaluate the strategies aimed at achieving the objectives established by the WHO in the global action plan for the prevention and control of non-communicable diseases [19].

With this vision, the SIMETAP-HTN study aims were to determine, in the adult population, the crude and adjusted prevalence rates of HTN according to the two established diagnostic criteria, to compare BP control in HTN patients with and without ASCVD or CKD according to both criteria, and to assess their associations with ASCVD and metabolic and renal factors.

## 2. Materials and Methods

The SIMETAP-HTN is sub-study from the SIMETAP study, whose methodology has been previously described [20], authorised by the Health Service of the Community of Madrid. In brief, it is a multicentre cross-sectional observational study, in which 121 physicians from 64 primary care centres participated. Simple random sampling of 5.45% of the target population aged 18 and over (194,073 adults) assigned to physicians was performed using random numbers drawn from the Excel function rand.between (bottom, top). Pregnant women, terminally ill patients or those with cognitive impairment, and institutionalised people were excluded per protocol. Six thousand five hundred and eighty-eight study subjects were selected after signing the informed consent and verifying that their medical records had the necessary clinical and laboratory data to be evaluated (response rate 62.9%). For the purposes of this study, HTN diagnosis (ICD-10-CM: I10, I15; ICPC-2: K86, K87) was considered according the following two criteria using the average of ≥2 BP readings obtained on ≥2 occasions or taking medication for HTN: 1. 140–90 criterion: SBP ≥ 140 mmHg and/or DBP ≥ 90 mmHg; 2. 130–80 criterion: SBP ≥ 130 mmHg and/or DBP ≥ 80 mmHg. The defining criteria of morbidities, variables, or clinical conditions assessed are shown in Table S1 (Supplementary Materials).

The Shapiro–Wilk test was used to check the data fitting to normal distribution for quantitative variables. If the variables showed normal distribution, they were analysed using mean, standard deviation (SD), and the Student’s t-test or analysis of variance. Median and interquartile range (IQR) of age were determined. Qualitative variables were analysed using percentages, Chi-square test, and odds ratios, with a 95% confidence interval (CI). The age- and sex-adjusted prevalence rates were calculated with the direct method, using standardised ten-year age groups and the information of the Spanish population as of January 2015 according to the National Institute of Statistics. To assess the individual effect of clinical conditions and CVRFs on dependent variable HTN, multivariate logistic regression analysis were performed using the backward stepwise method, initially introducing into the model all the variables that showed an association up to *p*-value < 0.10, except for metabolic syndrome (MetS) [21] because its definition integrates some factors assessed independently in the analysis, and for erectile dysfunction because it affects only men. Subsequently, the variable that contributed least to the fit of the analysis was eliminated at each step. A two-sided *p*-value < 0.05 was considered to indicate statistical significance. SPSS for Windows, version 25 (IBM, Armonk, NY, USA), was used for the statistical analysis. 

## 3. Results

The mean (SD) age was 55.1 (17.5), and the median (IQR) was 54.7 (41.7–68.1) years. Women represented 55.9% [95% CI: 54.7–57.1%]) of the sample. The median ages (IQR) were 55.0 (42.4–67.5) years in men and 54.5 (41.0–68.8) in women, with no significant differences (*p =* 0.634) in the mean ages (SD) among males (55.3 [16.9] years) and females (55.0 [18.0] years).

### 3.1. HTN Prevalence Rates According to 140/90 Criterion

**Figure 1:**
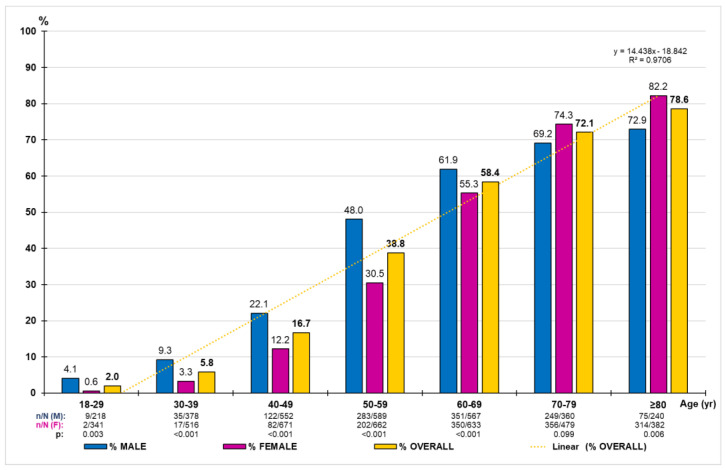
Prevalence rates of hypertension by age group according to 140/90 criterion. n: number of cases; N: sample size; M: male; F: female; p: *p*-value of the difference in percentages (M–F).

HTN crude prevalence was 38.7% (95% CI: 37.5–39.9%). The difference between males (42.2% [95%CI: 40.3–44.0%]) and females (35.9% [95% CI: 34.4–37.5%]) was significant (*p* < 0.001). The age- and sex-adjusted prevalence rate of HTN was 30.9% (32.9% male; 29.7% female). The HTN-adjusted prevalence rates were 35.0% (40.8% male; 29.9% female) in people between 40 and 69 years of age, and 75.0% (70.7% male; 77.8% female) in people older than 69 years of age. The distribution of the HTN prevalence rates by ten-year age groups increased precisely with age (R^2^ = 0.971) according to the linear function y = 14.438x − 18.842, being significantly higher in males than in females (*p* < 0.001), except in the 70s, where the difference was not significant (*p* = 0.099), and in the age group in the 80s, whose proportion of females was significantly higher than that of males (*p* = 0.006) (Figure 1). 

### 3.2. HTN Prevalence Rates According to 130/80 Criterion

**Figure 2:**
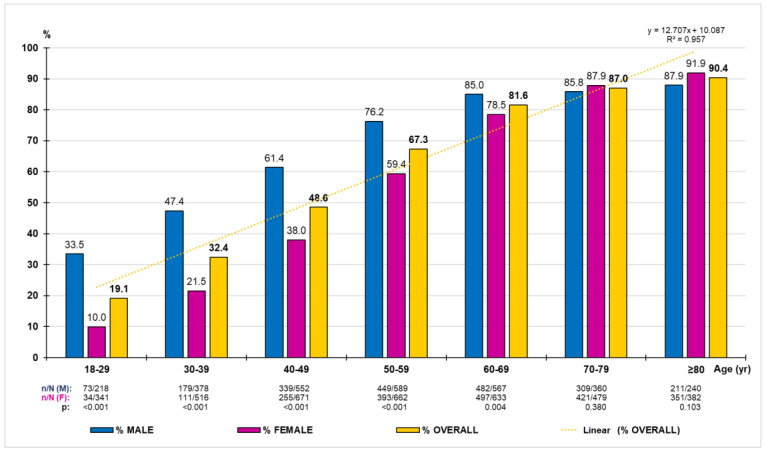
Prevalence rates of hypertension by age group according to 130/80 criterion. n: number of cases; N: sample size; M: male; F: female; *p*: *p*-value of the difference in percentages (M–F).

HTN crude prevalence was 62.3% (95% CI: 61.1–63.5%). The difference between males (70.3% [95% CI: 68.6–72.0%]) and females (56.0% [95% CI: 54.4–57.6%]) was significant (*p* < 0.001). The age- and sex-adjusted prevalence rate of HTN was 54.9% (63.2% male; 49.3% female). The HTN-adjusted prevalence rates were 63.5% (72.3% male; 58.2% female) in people between 40 and 69 years of age, and 88.5% (86.6% male; 89.8% female) in people older than 69 years of age. The distribution of the HTN prevalence rates by ten-year age groups increased precisely with age (R^2^ = 0.957) according to the linear function y = 12.707x − 10.087, being significantly higher in males than in females, except in the age groups from 70 onwards, in which there were no significant differences (Figure 2). 

### 3.3. Comparisons between Populations with and without HTN According to 140/90 Criterion

The median (IQR) ages of the populations with and without HTN were 67.5 (58.2–77.9) and 46.0 (36.4–57.2) years, respectively. The age means difference (19.7 years) between both populations was significant (*p* < 0.001) (Table 1). The rest of the quantitative clinical variables and the atherogenic and glycaemic indices were significantly higher (*p* < 0.001) in the population with HTN than in the population without HTN, except for total cholesterol, high-density lipoprotein cholesterol (HDL-C), and estimated glomerular filtration rate (eGFR), which were significantly (*p* < 0.001) higher in the population without HTN, and non-HDL-C and aspartate aminotransferase (AST), whose differences between both populations were not significant (Table 1).

### 3.4. Comparisons between Populations with and without HTN According to 130/80 Criterion

The median (IQR) ages of the populations with and without HTN were 62.2 (50.3–73.8) and 42.6 (33.4–53.9) years, respectively. The age means difference (17.0 years) between both populations being significant (*p* < 0.001) (Table 2). The rest of the quantitative clinical variables and the atherogenic and glycaemic indices were significantly higher in the population with HTN than in the population without HTN, except for HDL-C and eGFR, which were significantly higher (*p* < 0.001) in the population without HTN, and total cholesterol, low-density lipoprotein cholesterol (LDL-C) and AST, whose differences between both populations were not significant (Table 2).

### 3.5. Associations between HTN and CVRF, Renal and Cardiometabolic Diseases

All comorbidities and CVRF were significantly associated with HTN according to both criteria, except for smoking and the low and moderate CVR categories, which were significantly associated with populations without HTN (Table 3).

The following variables showed a strong association (OR between 3.0 and 6.0) with HTN diagnosed according to 140/90 criterion: obesity, abdominal obesity, increased waist-to-height ratio (WHtR), hypercholesterolemia, erectile dysfunction, and albuminuria. The variables diabetes mellitus (DM), atherosclerotic cardiovascular disease (ASCVD), CHD, stroke, peripheral arterial disease (PAD), atrial fibrillation (AF), low eGFR, CKD, and very high CVR showed a very strong association (OR > 6.0) with HTN, highlighting MetS (OR: 11.0) and heart failure (HF) (OR: 15.6). A total of 80.1% (95% CI: 78.5–81.6) of the HTN patients had a high or very high CVR (Table 3).

On the other hand, the following variables also showed a strong association with HTN diagnosed according to 130/80 criterion: obesity, abdominal obesity, high WHtR, DM, ASCVD, ictus, stroke, PAD, AF, erectile dysfunction, albuminuria, and CKD. The variables CHD, HF, low eGFR, and very high VR showed a very strong association (OR > 6.0) with HTN, highlighting MetS (OR: 11.4). A total of 62.8% (95% CI: 61.3–64.3) of the HTN patients had a high or very high CVR (Table 3).

### 3.6. Effect of Associated Comorbidities on HTN According to 140/90 or 130/80 Criteria

The comorbidities and clinical conditions that were best independently associated with HTN according to both criteria were HF, DM, CHD, low eGFR, and obesity. Other variables that were also independently associated with HTN according to both criteria were stroke, AF, hypercholesterolemia, hyperuricemia, high WHtR, prediabetes, and overweight. Albuminuria and PAD also showed an independent association with HTN, but only according to the 140/90 criterion (Table 4).

### 3.7. BP Control Targets in Patients with HTN Diagnosed According to Both Criteria

The percentages of patients without ASCVD or CKD diagnosed with HTN according to the 130/80 criterion on antihypertensive pharmacological treatment, and the mean number of BP-lowering drugs per patient was almost double that if they were diagnosed with the 140/90 criterion. There were small, although significant (*p* < 0.001), differences both in the percentage of HTN patients with BP control target < 140/90 mmHg or <130/80 mmHg, according to both diagnostic criteria (Table 5).

**Figure 3:**
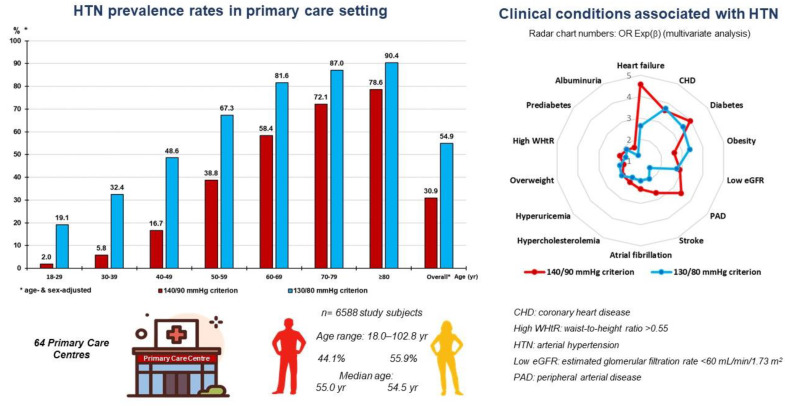
HTN prevalence rates according to 140/90 or 130/90 and related factors (graphical abstract).

On the other hand, the percentage of patients with ASCVD or CKD diagnosed with HTN according to the 130/80 criterion were significantly higher than according to the 140/90 criterion. The difference in percentage between patients on drug treatment diagnosed according to both criteria was not significant in patients with ASCVD (*p* = 0.058) and was slightly and significantly higher (*p* = 0.009) in patients with CKD. The mean daily number of drugs was similar, although significantly higher in patients diagnosed with ASCVD or CKD according to the 140/90 criterion. There were no significant differences in percentages of BP control targets < 140/90 mmHg or <130/80 mmHg according to both diagnostic criteria (Table 5). The main results are summarised in Figure 3.

## 4. Discussion

### 4.1. HTN Prevalence Rates

Most of the studies on HTN prevalence rates are based on the classic threshold SBP/DBP values 140/90 mmHg (16). According to these BP values, the age-standardised prevalence of HTN in high-income European countries was 20.6% (28.4% males; 15.9% females) [22]. Follow-ups carried out in the 1990s in Europe observed that BP increased with age, exceeding levels of 130/80 mmHg from the age of 45, and 140/90 mmHg from the age of 55 [23,24]. 

Age plays a fundamental role in the increase in BP throughout life and in determining the HTN prevalence rates. In our study, HTN prevalence rates increased with age with an almost perfect linear correlation using both diagnostic criteria. However, in the adult population, the adjusted prevalence of HTN defined according to the 140/90 criterion was 24.0% lower than according to the 130/80 criterion (54.9%); conversely, the mean age of the population with HTN defined according to the 140/90 criterion was 5.7 years older than the 130/80 criterion.

Considering the HTN diagnosis as BP levels ≥ 130/80 mmHg, the NHANES study showed than HTN prevalence was 46.7% in the adult population from the United States (50.4% males; 43.0% females) [25], 86.2% in subjects aged 75 or over, and 27.2% in the 20–44 age group [26]. In our study, almost two thirds of the population between 40 and 69 years of age (63.5%), and most of the people over 69 (88.5%) suffered from HTN according to the 130/80 criterion. When comparing the HTN-adjusted prevalence according to both criteria, it could be interpreted that 28.5% of people between 40 and 69 years of age, and 13.5% of those over 69, did not suffer from HTN if the diagnostic criterion 140/90 was used instead of the 130/80 criterion. In the adult population, the increase in the HTN-adjusted prevalence rates between the 140/90 and 130/80 criteria was greater in men (32.9% vs. 63.2%, respectively) than in women (29.8% vs. 49.3%, respectively).

Previous studies carried out in the Spanish population showed adjusted prevalence rates of HTN similar to the present study, such as the studies of Banegas et al. [27] (33.3%); ERICE [28] (37.6%); DARIOS [29] (43%); Dia@bet.es [30] (31.3% male, 28.6% female); Vega-Alonso et al. [31] (31.1%); PREDIMERC [32] (29.3%). The age- and sex-adjusted prevalence in the population ≥ 60 years of age in our study (67.5%) was similar to the study by Banegas et al. [14] (66.0%). However, other reports yielded lower results, such as the European Health Survey (EHS) [33] (18.4%), the Spanish National Health Survey (ENSE) [34] (19.8%), and the Clinical Database of Primary Care (BDCAP) of the Spanish Ministry of Health [35] (16.5%). These differences could be influenced by random selection biases, although it seems to be inferred that there is a significant percentage of the population that is unaware that they suffer from HTN.

### 4.2. Clinical Conditions and Factors Associated with HTN

In the adult population with HTN diagnosed according to the 140/90 criterion, the combined prevalence of overweight and obesity in our study was similar (84.4%) to that of the American population (84.3%) [36], although it differed when assessing overweight and obesity separately (41.3% and 43.4%, respectively, in our study, and 31.3% and 53.0%, respectively, in the American one). The high prevalence of a sedentary lifestyle and obesity in HTN patients makes it necessary to incorporate adequate lifestyles as adjuncts to the different pharmacological treatments. A high prevalence of other associated clinical conditions and CVRFs (prediabetes, DM, dyslipidaemia, MetS, ASCVD, and CKD) has also been observed in subjects with HTN. The presence of so many CVRFs also justifies the high prevalence of MetS in the present study, with both the 140/90 and 130/90 criteria (76.2% and 62.7%, respectively).

On the other hand, a strong association of HTN with both AF and HF was observed. High BP is the most important risk factor for HF [37] and the etiological factor most frequently associated with AF [38], so inadequate BP control can precipitate episodes of worsening CHD. All this can also justify the high percentage of hypertensive patients with high or very high CVR, with both the 140/90 and 130/90 criteria (80.1% and 62.7%, respectively).

The aggregation of CVRFs in patients with HTN is well known and increases their CVR [39]. It is important to know and assess the CVR of the patients as a whole. A Trialists’ meta-analysis [40] showed that the baseline CVR in patients with HTN was the greatest determinant of the absolute benefit of the treatment, emphasising the importance of using CVR equations to make decisions and that selecting patients based on their CVR to start treatment was more effective than treating them based on their BP. The study by Karmali at al. [41] showed in untreated hypertensive subjects that 64% of the events occurred in those with a CVR greater than 7.5% calculated with the pooled cohort risk equation. A secondary analysis of the Save Your Heart study [42] showed a very high probability of encountering a fatal or non-fatal cardiovascular event due to a lack of CVRF control in the hypertensive population. Therefore, better control of all CVRFs should be the main objective for the patient with HTN.

### 4.3. Comparison of BP Control Targets in Patients with HTN Diagnosed according to Both Criteria

The guidelines for HTN management [16,17] recommend starting BP-lowering medications in people without CHD or CKD, when the office SBP is ≥140 mmHg and/or DBP ≥ 90 mmHg. In our study, BP control < 140/90 of HTN patients without ASCVD or CKD was similar using both HTN diagnostic criteria (around 90%), although the percentage of patients treated with BP-lowering medications was almost twice with the 140/90 criterion than with the 130/80 criterion and using twice as many drugs per patient, respectively. Although the first goal of antihypertensive treatment should be to lower < 140/90 mmHg, additional effort should be made to achieve a BP range of 120–129/70–79 mmHg, if drug treatment is well tolerated [16,17]. The American and European guidelines agree on stricter control targets (<130/80 mmHg) in secondary prevention [16,17]. In primary prevention, new data have recently been published showing that SBP between 130 and 139 mm Hg has independent prognostic value in adults with low CVR and that these subjects could benefit from stricter SBP targets (<130 mm Hg) [43]. In our study, the achievement of BP levels < 130/80 mmHg decreased by almost 30 percentage points with respect to the percentage of BP control target < 140/90 using both diagnostic criteria for HTN (Table 5).

On the other hand, the recommended thresholds for BP-lowering drugs are ≥ 130/80 mmHg in patients with CHD or CKD (17,18). In our study, BP control target < 130/80 in HTN patients with ASCVD or CKD was similar using both HTN diagnostic criteria (around 70%), although the percentage of patients treated with BP-lowering medications and the mean number of antihypertensive drugs per patient were slightly higher using the 140/90 criterion than with the 130/90 criterion. 

### 4.4. Strengths and Limitations

The study limitations were the sampling bias due to the recruitment response rate, the inability to estimate incidence rates or to determine causality, inter-interviewer variability, possible heterogeneity of the measurement and laboratory equipment, and the HTN underdiagnoses due to the per protocol exclusion of terminally ill, institutionalised, or cognitively impaired patients and pregnant women. BP control of HTN patients was excellent, probably due to the study selection bias of excluding subjects without the clinical or laboratory data necessary to be evaluated, who are the subjects with the worst follow-up. Strengths of the study include a large population-based random sample aged 18.0–102.8, the determination of both crude and adjusted prevalence rates of HTN according to two BP threshold values defined by main international guidelines for the HTN management, and the assessment of the association of HTN with renal and cardiometabolic factors.

### 4.5. Clinical Implications

The high prevalence of HTN has serious healthcare and socioeconomic consequences by increasing cardiovascular morbidity and mortality. Assessing the epidemiological magnitude of HTN is essential to better plan prevention policies aimed at reducing the HTN burden, to improve medical care and quality of life for patients, and to optimise available health resources. In order to compare HTN prevalence rates with other populations, they should always be age-adjusted because HTN is strongly associated with increasing age.

The early identification of HTN patients has some important implications. In our study, almost two thirds of the population with HTN diagnosed according to the 130/80 criterion had a high or very high CVR; hence, the early identification of these patients would facilitate starting a comprehensive management not only of BP but also of other comorbidities and clinical conditions that are frequently associated with HTN. In addition, the implementation of the 130/80 diagnostic criterion would imply that almost a quarter more of the HTN population could be identified almost 6 years earlier and start counselling on changes to healthy lifestyles and pharmacological treatment when indicated, in order to delay the increase in BP, decrease CVR, and avoid cardiovascular complications.

We hope that this study updates the knowledge of the HTN prevalence rates and contributes to emphasising the importance and magnitude of the clinical conditions and comorbidities that are associated with HTN to promote the comprehensive diagnostic and therapeutic management of this disease.

## 5. Conclusions

Almost a third of the adult population with HTN were diagnosed according to the 140/90 criterion and more than half according to the 130/80 criterion. The use of the 130/80 criterion for HTN diagnosis compared to the 140/90 criterion means that the HTN prevalence rates increase almost 20 percentage points from 29.7% in women and more than 30 percentage points from 32.9% in men. The distribution of the HTN prevalence rates increases linearly with age using both criteria.

The intensity of BP-lowering drug treatment and the proportion of HTN patients achieving the BP control target < 130/80 mmHg was similar in both ASCVD and CKD patients, regardless of the diagnostic criteria used. The clinical conditions that were most strongly associated with HTN according to both diagnostic criteria were HF, DM, CHD, low eGFR, and obesity. Stroke and PAD also maintain a strong association with HTN according to the 140/90 criterion. The proportion of HTN patients with elevated CVR was higher in those diagnosed according to the 140/90 criterion (80.1%) than in those diagnosed according to the 130/80 criterion (62.8%). The high cardiovascular burden of HTN justifies the need to implement measures to achieve the BP control objectives recommended by the guidelines and to optimise the comprehensive management of clinical conditions and comorbidities associated with HTN.

### Key Points

WHAT IS KNOWN ABOUT THE TOPIC?

- The proportion of adults with HTN doubled between 1990 and 2019 worldwide.
- Primary health care is the setting where HTN is usually detected.
- There is a continuous relationship between BP and cardiovascular or renal morbid or fatal events starting from SBP/DBP values >115/75 mmHg.
- The American guidelines recommend that the threshold values for SBP/DBP to consider HTN should be 130/80 mmHg, which are different from the classic threshold values (140/90 mmHg) recommended according to other international guidelines for the HTN management.
- The discrepancy with these threshold levels raises differences, not only diagnostic, but also in the determination of the HTN prevalence and in the assessment of the clinical conditions and comorbidities that can be associated with HTN.
- Early initiation of antihypertensive treatment can effectively control hypertension and prevent its progression and associated cardiovascular, metabolic, and renal complications.

WHAT DOES THIS STUDY ADD?

- The mean age of the HTN patients diagnosed according to the 140/90 criterion was 5.7 years older than those diagnosed according to the 130/80 criterion (61.5 years).
- The age- and sex-adjusted prevalence rate of HTN was 30.9% according to the 140/90 criterion and was 54.9% according to the 130/80 criterion.
- The proportion of HTN patients with high or very high CVR was higher in those diagnosed according to the 140/90 criterion (80.1%) than in those diagnosed according to the 130/80 criterion (62.8%).
- The proportion of HTN patients without ASCVD or CKD who achieved the BP control goal < 130/80 mmHg was 60.5% in those diagnosed according to the 140/90 criterion and 65.5% according to the 130/80 criterion.
- The proportion of HTN patients according to both diagnostic criteria who achieved the BP control target < 130/80 mmHg was similar in patients with ASCVD (70%) and in patients with CKD (71%).
- The intensity of treatment with BP-lowering drugs in HTN patients without ASCVD or CKD decreased by half when using the 130/80 diagnostic criterion than when using the 140/90 criterion, and instead, it was similar for both HTN patients with ASCVD and with CKD.
- The following comorbidities and clinical conditions showed, from greater to lesser intensity, an independent association with HTN according to both criteria: HF, DM, CHD, low eGFR, obesity, stroke, AF, hypercholesterolemia, hyperuricemia, high WHtR, prediabetes, and overweight. Albuminuria and PAD showed an independent association only with HTN according to the 140/90 criterion.

## Figures and Tables

**Table 1:** Clinical characteristics of populations according to hypertension diagnostic criterion 140/90.

	with Hypertension		without Hypertension		Difference in Means	*p*
	N	Mean (SD)	N	Mean (SD)		
Age (year)	2547	67.2 (13.3)	4041	47.5 (15.5)	19.7	<0.001
Body mass index (kg/m^2^)	2547	29.8 (5.0)	4041	26.1 (4.7)	3.7	<0.001
Abdominal circumference (cm)	2547	99.9 (13.0)	4041	89.2 (13.1)	10.7	<0.001
WHtR	2547	0.62 (0.08)	4041	0.54 (0.08)	0.08	<0.001
SDB (mmHg)	2547	130.8 (15.3)	4041	116.4 (12.7)	14.4	<0.001
DBP (mmHg)	2547	76.6 (9.8)	4041	71.3 (9.2)	5.3	<0.001
Pulse pressure (mmHg)	2547	54.2 (13.1)	4041	45.0 (9.6)	9.2	<0.001
FPG (mg/dL) ^a^	2547	105.8 (30.8)	4041	89.8 (20.0)	16.0	<0.001
HbA1c (%) ^b^	2192	6.0 (1.0)	3041	5.4 (0.7)	0.6	<0.001
Total cholesterol (mg/dL) ^c^	2547	190.3 (38.9)	4041	194.3 (39.6)	−4.0	<0.001
HDL-C (mg/dL) ^c^	2547	53.2 (14.8)	4041	55.8 (14.5)	−2.6	<0.001
Non-HDL-C (mg/dL) ^c^	2547	137.1 (37.1)	4041	138.5 (41.4)	−1.4	0.157
LDL-C (mg/dL) ^c^	2530	110.9 (34.3)	3996	116.2 (34.5)	−5.3	<0.001
VLDL-C (mg/dL) ^c^	2530	25.6 (12.8)	3996	21.2 (11.7)	4.4	<0.001
Triglycerides (mg/dL) ^d^	2547	132.0 (73.2)	4041	113.3 (88.2)	18.7	<0.001
Non-HDL-C / HDL-C	2547	2.8 (1.1)	4041	2.7 (1.2)	0.1	0.001
Triglycerides / HDL-C	2547	2.8 (2.2)	4041	2.3 (2.7)	0.5	<0.001
TyG index	2547	8.7 (0.6)	4041	8.4 (0.6)	0.3	<0.001
Uric acid (mg/dL) ^e^	2435	5,4 (1.5)	3733	4.6 (1.4)	0.8	<0.001
AST (U/L)	1913	24.4 (49.1)	2899	22.1 (38.7)	2.3	0.073
ALT (U/L)	2470	25.7 (17.5)	3943	24.3 (16.5)	1.4	0.001
GGT (U/L)	2352	39.0 (52.0)	3723	29.8 (49.8)	9.2	<0.001
Creatinine (mg/dL) ^f^	2547	0.90 (0.38)	4041	0.80 (0.22)	0.10	<0.001
eGFR (mL/min/1.73 m^2^)	2547	79.6 (19.8)	4041	97.5 (17.8)	−17.9	<0.001
uACR (mg/g) ^g^	2547	26.9 (88.8)	4041	9.8 (29.6)	17.1	<0.001

ALT: alanine aminotransferase; AST: aspartate aminotransferase; DBP: diastolic blood pressure; eGFR: estimated glomerular filtration rate; FPG: fasting plasma glucose; GGT: gamma-glutamyl transferase; HbA1c: glycated haemoglobin A1c; HDL-C: high-density lipoprotein cholesterol; LDL-C: low-density lipoprotein cholesterol; SBP: systolic blood pressure; TyG index: triglyceride and glucose index; uACR: urine albumin–creatinine ratio; VLDL-C: very low-density lipoprotein cholesterol; WHtR: waist-to-height ratio. The definitions of the variables are shown in Table S1 (Supplementary Materials). *p*: *p*-value of difference in means; ^a^ to convert from mg/dL to mmol/L, multiply by 0.05556; ^b^ to convert from % (DCCT) to mmol/mol (IFCC), multiply by 0.09148 and add 2.152; ^c^ to convert from mg/dL to mmol/L, multiply by 0.02586; ^d^ to convert from mg/dL to mmol/L, multiply by 0.01129; ^e^ to convert from mg/dL to mmol/L, multiply by 0.05948; ^f^ to convert from mg/dL to mmol/L, multiply by 0.08842; ^g^ to convert from mg/g to mg/mmol, multiply by 0.01131.

**Table 2:** Clinical characteristics of populations according to hypertension diagnostic criterion 130/80.

	with Hypertension		without Hypertension		Difference in Means	*p*
	N	Mean (SD)	N	Mean (SD)		
Age (year)	4104	61.5 (15.7)	2484	44.5 (15.1)	17.0	<0.001
Body mass index (kg/m^2^)	4104	29.0 (5.0)	2484	25.0 (4.3)	4.0	<0.001
Abdominal circumference (cm)	4104	97.8 (13.2)	2484	86.1 (12.4)	11.7	<0.001
WHtR	4104	0.60 (0.08)	2484	0.52 (0.08)	0.08	<0.001
SDB (mmHg)	4104	129.2 (13.4)	2484	109.9 (10.2)	19.3	<0.001
DBP (mmHg)	4104	76.6 (9.8)	2484	71.3 (9.2)	5.3	<0.001
Pulse pressure (mmHg)	4104	51.5 (12.8)	2484	43.8 (8.5)	7.7	<0.001
FPG (mg/dL) ^a^	4104	101.3 (29.2)	2484	87.3 (16.1)	14.0	<0.001
HbA1c (%) ^b^	3403	5.8 (1.0)	1830	5.3 (0.6)	0.5	<0.001
Total cholesterol (mg/dL) ^c^	4104	193.4 (38.6)	2484	191.7 (40.5)	1.7	0.082
HDL-C (mg/dL) ^c^	4104	53.4 (14.5)	2484	57.1 (14.7)	−3.7	<0.001
Non-HDL-C (mg/dL) ^c^	4104	140.0 (37.3)	2484	134.5 (40.0)	5.5	<0.001
LDL-C (mg/dL) ^c^	4066	114.4 (34.0)	2460	113.6 (35.4)	0.8	0.388
VLDL-C (mg/dL) ^c^	4066	24.8 (12.7)	2460	19.8 (10.9)	5.0	<0.001
Triglycerides (mg/dL) ^d^	4104	129.9 (83.7)	2484	105.1 (80.0)	24.8	<0.001
Non-HDL-C / HDL-C	4104	2.8 (1.1)	2484	2.5 (1.1)	0.3	<0.001
Triglycerides / HDL-C	4104	2.8 (2.6)	2484	2.1 (2.5)	0.7	<0.001
TyG index	4104	8.6 (0.6)	2484	8.2 (0.6)	0.4	<0.001
Uric acid (mg/dL) ^e^	3870	5.2 (1.5)	2298	4.4 (1.4)	0.8	<0.001
AST (U/L)	3030	23.9 (39.9)	2899	21.6 (48.1)	2.3	0.072
ALT (U/L)	3998	26.4 (18.6)	3943	22.4 (13.3)	4.0	<0.001
GGT (U/L)	3782	37.9 (59.4)	2293	26.0 (30.6)	11.9	<0.001
Creatinine (mg/dL) ^f^	4104	0.88 (0.32)	2484	0.78 (0.22)	0.10	<0.001
eGFR (mL/min/1.73 m^2^)	4104	84.8 (20.0)	2484	99.9 (17.7)	−15.1	<0.001
uACR (mg/g) ^g^	4104	20.9 (74.8)	2484	9.0 (18.8)	11.9	<0.001

ALT: alanine aminotransferase; AST: aspartate aminotransferase; DBP: diastolic blood pressure; eGFR: estimated glomerular filtration rate; FPG: fasting plasma glucose; GGT: gamma-glutamyl transferase; HbA1c: glycated haemoglobin A1c; HDL-C: high-density lipoprotein cholesterol; LDL-C: low-density lipoprotein cholesterol; SBP: systolic blood pressure; TyG index: triglyceride and glucose index; uACR: urine albumin–creatinine ratio; VLDL-C: very low-density lipoprotein cholesterol; WHtR: waist-to-height ratio. The definitions of the variables are shown in Table S1 (Supplementary Materials). *p*: *p*-value of difference in means; ^a^ to convert from mg/dL to mmol/L, multiply by 0.05556; ^b^ to convert from % (DCCT) to mmol/mol (IFCC), multiply by 0.09148 and add 2.152; ^c^ to convert from mg/dL to mmol/L, multiply by 0.02586; ^d^ to convert from mg/dL to mmol/L, multiply by 0.01129; ^e^ to convert from mg/dL to mmol/L, multiply by 0.05948; ^f^ to convert from mg/dL to mmol/L, multiply by 0.08842; ^g^ to convert from mg/g to mg/mmol, multiply by 0.01131.

**Table 3:** Association of clinical conditions and comorbidities with HTN according to 140/90 and 130/80 criteria.

	with HTN 140/90 (%) N = 2547	without HTN 140/90 (%) N = 4041	OR (95% CI)	with HTN 130/80 (%) N = 4104	without HTN 130/80 (%) N = 2484	OR (95% CI)
Male	1224 (48.1)	1680 (41.6)	1.3 (1.2–1.4)	2042 (49.8)	862 (34.7)	1.9 (1.7–2.1)
Current smoking	395 (15.5)	1031 (25.5)	0.5 (0.5–0.6)	811 (19.8)	615 (24.8)	0.7 (0.7–0.8)
Physical inactivity	1281 (50.3)	1798 (44.5)	1.3 (1.1–1.4)	2007 (48.9)	1072 (43.2)	1.3 (1.1–1.4)
Overweight	1053 (41.3)	1463 (36.2)	1.2 (1.1–1.4)	1708 (41.6)	808 (32.5)	1.5 (1.3–1.6)
Obesity	1104 (43.4)	729 (18.0)	3.5 (3.1–3.9)	1531 (37.3)	302 (12.2)	4.3 (3.8–4.9)
Abdominal obesity	1614 (63.4)	1308 (32.4)	3.6 (3.3–4.0)	2293 (55.9)	629 (25.3)	3.7 (3.4–4.2)
High WHtR	2018 (79.2)	1678 (41.5)	5.4 (4.8–6.0)	2889 (70.4)	807 (32.5)	4.9 (4.4–5,5)
Prediabetes	754 (29.6)	695 (17.2)	2.0 (1.8–2.3)	1112 (27.1)	337 (13.6)	2.4 (2.1–2.7)
Diabetes	771 (30.3)	264 (6.5)	6.2 (5.3–7.2)	917 (22.3)	118 (4.8)	5.8 (4.7–7.0)
Hypercholesterolemia	2002 (78.6)	2099 (51.9)	3.4 (3.0–3.8)	2942 (71.7)	1159 (446.7)	2.9 (2.6–3.2)
Low HDL-C	856 (33.6)	963 (23.8)	1.6 (1.5–1.8)	1272 (31.0)	547 (22.0)	1.6 (1.4–1.8)
Hypertriglyceridemia	1008 (39.6)	939 (23.2)	2.2 (1.9–2.4)	1469 (35.8)	478 (19.2)	2.3 (2.1–2.6)
Atherogenic dyslipidaemia	522 (20.5)	419 (10.4)	2.2 (2.0–2.6)	737 (18.0)	204 (8.2)	2.4 (2.1–2.9)
Hyperuricemia	562 (21.6)	257 (6.9)	3.7 (3.2–4.7)	670 (17.3)	113 (4.9)	4.0 (3.3–5.0)
Metabolic syndrome	1941 (76.2)	910 (22.5)	11.0 (9.8–12.4)	2541 (61.9)	310 (12.5)	11.4 (10.0–13.0)
CHD	269 (10.6)	52 (1.3)	9.1 (6.7–12.2)	299 (7.3)	22 (0.9)	8.8 (5.7–13.6)
Stroke	196 (7.7)	54 (1.3)	6.2 (4.5–8.4)	219 (5.3)	31 (1.2)	4.5 (3.1–6.5)
PAD	126 (4.9)	24 (0.6)	8.7 (5.6–13.5)	133 (3.2)	17 (0.7)	4.9 (2.9–8.1)
ASCVD	493 (19.4)	122 (3.0)	7.7 (6.3–9.5)	549 (13.4)	66 (2.7)	5.7 (4.4–7.3)
Heart failure	166 (6.5)	18 (0.4)	15.6 (9.6–25.4)	172 (4.2)	12 (0.5)	9.0 (5.0–16.2)
Atrial fibrillation	202 (7.9)	48 (1.2)	7.2 (5.2–9.9)	225 (5.5)	25 (1.0)	5.7 (3.8–8.7)
Erectile dysfunction ^a^	363 (29.7)	141 (8.4)	3.5 (3.0–4.2)	433 (21.2)	71 (8.2)	3.0 (2.3–3.9)
Albuminuria	291 (11.4)	103 (2.6)	4.9 (3.9–6.2)	336 (8.2)	58 (2.3)	3.7 (2.8–5.0)
Low eGFR	426 (16.7)	97 (2.4)	8.2 (6.5–10.2)	479 (11.7)	44 (1.8)	7.3 (5.4–10.0)
CKD	581 (22.8)	175 (4.3)	6.5 (5.5–7.8)	668 (16.3)	88 (3.5)	5.3 (4.2–6.6)
Low CVR	104 (4.1)	2041 (50.5)	0.04 (0.03–0.05)	607 (14.8)	1538 (61.9)	0.11 (0.09–0.12)
Moderate CVR	404 (15.9)	975 (24.1)	0.6 (0.5–0.7)	921 (22.4)	458 (18.4)	1.2 (1.1–1.4)
High CVR	550 (21.6)	473 (11.7)	2.1 (1.8–2.4)	798 (19.4)	225 (9.1)	2.4 (2.1–2.8)
Very high CVR	1489 (58.5)	552 (13.7)	8.9 (7.9–10.0)	1778 (43.3)	263 (10.6)	6.5 (5.6–7.4)

^a^ N: sample size (male): with HTN 140/90: 1224; without HTN 140/90: 1680; with HTN 130/80: 2042; without HTN 130/80: 862. ASCVD: atherosclerotic cardiovascular disease; CHD: coronary heart disease; CKD: chronic kidney disease; CVR: cardiovascular risk; eGFR: estimated glomerular filtration rate; HDL-C: high-density lipoprotein cholesterol; HTN: arterial hypertension; PAD: peripheral arterial disease; WHtR: waist-to-height ratio. The definitions of the comorbidities or clinical conditions are shown in Table S1 (Supplementary Materials).

**Table 4:** Effect of clinical conditions and comorbidities on HTN according to 140/90 and 130/80 criteria.

HTN 140/90	β ^a^	OR Exp(β) ^b^	*p* ^c^	HTN 130/80	β ^a^	OR Exp(β) ^b^	*p* ^c^
Heart failure	1.52 (0.31)	4.57 (2.52–8.30)	<0.001	CHD	1.31 (0.26)	3.71 (2.23–6.17)	<0.001
Diabetes	1.38 (0.09)	3.97 (3.31–4.77)	<0.001	Diabetes	1.26 (0.12)	3.54 (2.83–4.44)	<0.001
CHD	1.28 (0.18)	3.61 (2.51–5.17)	<0.001	Obesity	1.21 (0.11)	3.36 (2.72–4.15)	<0.001
PAD	1.24 (0.27)	3.44 (2.04–5.81)	<0.001	Low eGFR	1.01 (0.18)	2.75 (1.95–3.88)	<0.001
Low eGFR	1.06 (0.14)	2.89 (2.20–3.78)	<0.001	Heart failure	0.96 (0.36)	2.62 (1.29–5.33)	0.008
Stroke	0.99 (0.19)	2.68 (1.85–3.89)	<0.001	Hyperuricemia	0.76 (0.12)	2.13 (1.70–2.68)	<0.001
Obesity	0.96 (0.11)	2.62 (2.11–3.26)	<0.001	Overweight	0.69 (0.08)	2.00 (1.72–2.33)	<0.001
Atrial fibrillation	0.85 (0.20)	2.34 (1.58–3.49)	<0.001	Atrial fibrillation	0.67 (0.25)	1.94 (1.19–3.18)	0.008
Hypercholesterolemia	0.77 (0.07)	2.16 (1.89–2.48)	<0.001	Stroke	0.66 (0.22)	1.94 (1.25–3.00)	0.003
Hyperuricemia	0.73 (0.10)	2.07 (1.71–2.50)	<0.001	Hypercholesterolemia	0.63 (0.06)	1.88 (1.66–2.12)	<0.001
High WHtR	0.69 (0.09)	1.99 (1.68–2.36)	<0.001	Prediabetes	0.61 (0.08)	1.83 (1.57–2.14)	<0.001
Prediabetes	0.60 (0.08)	1.83 (1.58–2.11)	<0.001	High WHtR	0.54 (0.08)	1.71 (1.46–2.00)	<0.001
Overweight	0.60 (0.09)	1.82 (1.51–2.18)	<0.001	PAD *	0.43 (0.29)	1.54 (0.87–2.73)	0.136
Albuminuria	0.51 (0.15)	1.67 (1.25–2.24)	0.001	Albuminuria *	0.24 (0.17)	1.27 (0.90–1.78)	0.169

^a^ β coefficient (deviation); ^b^ odds-ratio Exp (β) (95% confidence interval); ^c^ *p*: *p*-value of Wald test with one degree of freedom; * not independently associated with HTN; CHD: coronary heart disease; Low eGFR: estimated glomerular filtration rate < 60 mL/min/1.73 m^2^; high WHtR: waist-to-height ratio > 0.55; HTN: arterial hypertension; PAD: peripheral arterial disease.

**Table 5:** BP control targets in HTN patients with and without ASCVD or CKD.

	without ASCVD or CKD			with ASCVD			with CKD		
	140/90 Criterion	130/80 Criterion	*p*	140/90 Criterion	130/80 Criterion	*p*	140/90 Criterion	130/80 Criterion	*p*
HTN patients *	1652 (30.5)	3074 (56.8)	<0.001	493 (80.2)	549 (89.3)	<0.001	581 (76.9)	668 (88.4)	<0.001
With BP < 140/90 mmHg *	1472 (89.1)	2846 (92.6)	<0.001	455 (92.3)	509 (92.7)	0.797	535 (92.1)	618 (92.5)	0.776
With BP < 130/80 mmHg *	999 (60.5)	2013 (65.5)	<0.001	343 (69.6)	386 (70.3)	0.795	412 (70.9)	472 (70.7)	0.923
On BP-lowering drugs *	1380 (83.5)	1420 (46.2)	<0.001	459 (93.1)	493 (89.8)	0.058	532 (91.6)	581 (87.0)	0.009
Daily BP-lowering drugs ^§^	1.40 (0.93)	0.77 (0.97)	<0.001	2.19 (1.15)	2.01 (1.23)	0.015	1.96 (1.10)	1.73 (1.20)	0.001

* N (%); ^§^ mean (SD): daily number of BP-lowering drugs per patient; *p*: *p*-value of difference in percentages or means; ASCVD: atherosclerotic cardiovascular disease; BP: blood pressure; CKD: chronic kidney disease; HTN: arterial hypertension.

## Data Availability

Data sharing is not applicable to this study.

## Supplementary Materials

The following supporting information can be downloaded at https://www.mdpi.com/article/10.3390/medicina59101846/s1, Table S1: Defining criteria of morbidities, variables, or clinical conditions assessed. Refs. [44,45,46,47,48,49,50] are cited in the supplementary materials.

## Abbreviations

AF	atrial fibrillation
ASCVD	atherosclerotic cardiovascular diseases
AST	aspartate aminotransferase
BP	blood pressure
CHD	coronary heart disease
CKD	chronic kidney disease
CVR	cardiovascular risk
CVRF	cardiovascular risk factors
DBP	diastolic blood pressure
DM	diabetes mellitus
eGFR	estimated glomerular filtration rate
HDL-C	high-density lipoprotein cholesterol
HF	heart failure
HTN	arterial hypertension
LDL-C	low-density lipoprotein cholesterol
MetS	metabolic syndrome
PAD	peripheral arterial disease
SBP	systolic blood pressure
WHtR	waist-to-height ratio
