# Supplementary material for: Prevalence Rates of Arterial Hypertension According to the Threshold Criteria of 140/90 or 130/80 mmHg and Associated Cardiometabolic and Renal Factors: SIMETAP-HTN Study
Source: Medicina (Kaunas). 2023 Oct 17;59(10):1846. doi: 10.3390/medicina59101846 (PMC10608132; doi:10.3390/medicina59101846)
Supplement: Supplementary file 1 [file medicina-59-01846-s001.zip › medicina-2619005-supplementary.pdf]

SUPPLEMENTARY MATERIALS

Table S1

Defining criteria of morbidities, variables or clinical conditions assessed

**Morbidities, variables or clinical conditions**	**Defining criteria**
Current smoking	Any amount of tobacco use in the past year.
Alcoholism	>21 standard drink units (SDU) of alcohol consumption (1 SDU= 10 g) per week (male), >14 SDU per week (female)
Physical inactivity	Physical activity <150 minutes per week
Overweight (1)	Body mass index (BMI) 25.0–29.9 kg/m^2 (ICD-10-CM: E66.3; ICPC-2: T83)*
Obesity (1)	BMI ≥30 kg/m^2 (ICD-10-CM: E66.9; ICPC-2: T82)*
Abdominal or central obesity (2)	Increased waist circumference (≥102 cm [male]; ≥88 cm [female]) determined with the subject standing using a flexible tape measure adjusted without compressing the skin, at the end of a normal expiration, locating the upper edge of the iliac crests and above that point surrounding the waist parallel to the floor.
High waist-to-height ratio (WHtR) (3)	Waist circumference/height ≥ 0.55
Arterial hypertension (HTN)	140/90 criteria (4): Systolic blood pressure (SBP) ≥140 mmHg and/or diastolic blood pressure (DBP) ≥90 mmHg, using the average of ≥2 readings obtained on ≥2 occasions, or being on antihypertensive treatment (ICD-10-CM: I10, I15; ICPC-2: K86, K87)*
	130/80 criteria (5): SBP ≥130 mmHg and/or DBP ≥80 mmHg, using the average of ≥2 readings obtained on ≥2 occasions, or being on antihypertensive treatment (ICD-10-CM: I10, I15; ICPC-2: K86, K87)*
Pulse pressure	SBP – DBP (mmHg)
Diabetes mellitus (DM)	According to the American Diabetes Association (ADA) criteria (6): fasting plasma glucose (FPG) ≥126 mg/dL or glycated hemoglobin A1c (HbA1c) ≥6.5%, or plasma glucose ≥ 200 mg/dL at any time or with oral glucose tolerance test (ICD-10-CM: E10, E11; ICPC-2: T89, T90)*
Prediabetes	According to the ADA criteria (6): FPG between 100 and 125 mg/dL or HbA1c between 5.7% and 6.4% (ICD-10-CM: R73.09; ICPC-2: A91)*
Hypercholesterolaemia	Total cholesterol (TC) ≥200 mg/dL (ICD-10-CM: E78; ICPC-2: T93)*
Hypertriglyceridaemia	Triglycerides (TG) ≥150 mg/dL (≥1.7 mmol/L) (ICD-10-CM: E78; ICPC-2: T93)*
Low high-density lipoprotein cholesterol (HDL-C)	HDL-C <40 mg/dL (<1.03 mmol/L) (male)<br>HDL-C <50 mg/dL (<1.29 mmol/L) (female)

Non-high-density lipoprotein cholesterol (Non-HDL-C)	TC – HDL-C
Low-density lipoprotein cholesterol (LDL-C)	TC – HDL-C – (TG/5) in mg/dL (not valid for patients with TG >400 mg/dL)
Very low-density lipoproteins cholesterol and remnants (VLDL-C)	TC – HDL-C – LDL-C
Triglyceride-glucose index (TyG)	Ln (TG x FPG/2)
Atherogenic dyslipidaemia	Hypertriglyceridaemia and low HDL-C
Metabolic syndrome (MetS)	According to Harmonized Consensus of International Diabetes Federation task force on Epidemiology and Prevention, National Heart, Lung, and Blood Institute, American Heart Association, World Heart Federation, International Atherosclerosis Society, and International Association for the Study of Obesity (2):<br>At least, three of following factors for the European population:<br>• Increased waist circumference (≥102 cm [male]; ≥88 cm [female])<br>• FPG ≥100 mg/dL (≥5.6 mmol/L)<br>• TG ≥150 mg/dL (≥1.7 mmol/L)<br>• HDL-C <40 mg/dL (<1.03 mmol/L) (males); <50 mg/dL (<1.29 mmol/L) (females)<br>• SBP ≥130 mmHg or DBP ≥85 mmHg or antihypertensive treatment
Hyperuricemia	Uric acid >7 mg/dL (male), >6 mg/dL (female) (ICD-10-CM: E79; ICPC-2: T92)*
Coronary heart disease (CHD)	Ischemic heart disease, acute myocardial infarction, acute coronary syndrome, coronary revascularization (ICD-10-CM: I20-I25; ICPC-2: K74, K75, K76)*
Cerebrovascular disease (stroke)	Cerebral ischemia, intracranial haemorrhage, transient ischemic attack (ICD-10-CM: I60-I66, I66, I67; ICPC-2: K89, K90K K91)*
Peripheral arterial disease (PAD)	Intermittent claudication, ankle-brachial index ≤ 0.9 (ICD-10-CM: I70.2, I73.9; ICPC-2: K92)*
Atherosclerotic vascular disease (ASCVD)	CHD, stroke, PAD (ICD-10-CM: I70)*
Heart failure (HF)	Record of the HF diagnosis in the clinical history (ICD-10-CM: I50; ICPC-2: K77)*
Atrial fibrillation (AF)	Record of the AF diagnosis in the clinical history (ICD-10-CM: I48; ICPC-2: K78)*

Estimated glomerular filtration rate (eGFR)	According to Chronic Kidney Disease Epidemiology Collaboration (CKD-EPI) equations (7):<br>Women with creatinine ≤ 0,7 mg/dL= 144 x $(creatinine)^{-0,329}$ x $(0,993)^{age}$ mL/min/1.73 m^2<br>Women with creatinine > 0,7 mg/dL= 144 x $(creatinine)^{-1,209}$ x $(0,993)^{age}$ mL/min/1.73 m^2<br>Men with creatinine ≤ 0,9 mg/dL= 141 x $(creatinine)^{-0,411}$ x $(0,993)^{age}$ mL/min/1.73 m^2<br>Men with creatinine > 0,9 mg/dL= 141 x $(creatinine)^{-1,209}$ x $(0,993)^{age}$ mL/min/1.73 m^2
Low eGFR (8)	eGFR <60 mL/min/1.73 m^2 according to CKD-EPI
Albuminuria (8)	Urine albumin-creatinine ratio (ACR) ≥30 mg/g (including proteinuria [ACR >300 mg/g] (ICD-10-CM: R80; ICPC-2: U98)*
Chronic kidney disease (CKD) (8)	Low eGFR and/or albuminuria (ICD-10-CM: N18; ICPC-2: U99)*
Cardiovascular risk (CVR) categories	Low, moderate, high and very high CVR according to SCORE *(systematic coronary risk evaluation)* (9) or SCORE-OP (10) for low-risk European countries (9)

* National Center for Health Statistics (NCHS). International Classification of Diseases, Tenth Revision, Clinical Modification (ICD-10-CM). [Accessed August 31, 2023]. Available from: https://www.cdc.gov/nchs/icd/icd-10-cm.htm#print

* World Health Organization. (2009). International Classification of Primary Care, -ICPC-2. [Accessed August 31, 2023]. Available from: https://www.who.int/standards/classifications/other-classifications/international-classification-of-primary-care

References

1. WHO Consultation on Obesity (1999: Geneva, Switzerland) & World Health Organization. (2000). Obesity: preventing and managing the global epidemic: Report of a WHO consultation. WHO technical report series; 894. [Accessed August 31, 2023]. Available from: https://apps.who.int/iris/handle/10665/42330.
2. Alberti KGMM, Eckel RH, Grundy SM, Zimmet PZ, Cleeman JI, Donato KA, et al. Harmonizing the metabolic syndrome: A joint interim statement of the International Diabetes Federation task force on Epidemiology and Prevention; National Heart, Lung, and Blood Institute; American Heart Association; World Heart Federation; International Atherosclerosis Society; and International Association for the Study of Obesity. Circulation. 2009;120:1640–5, https://doi.org/10.1161/CIRCULATIONAHA.109.192644.
3. Romero-Saldaña M, Fuentes-Jiménez FJ, Vaquero-Abellán M, Álvarez-Fernández C, Aguilera-López MD, Molina-Recio G. Predictive capacity and cutoff value of waist-to-height ratio in the incidence of metabolic syndrome. Clin Nurs Res. 2019;28:676–91, https://doi.org/10.1177/1054773817740533.
4. Mancia G, Kreutz R, Brunström M, Burnier M. Grassi G, Januszewicz A, et al. 2023 ESH Guidelines for the management of arterial hypertension. The Task Force for the management of arterial hypertension of the European Society of Hypertension. Endorsed by the European Renal Association (ERA) and the International Society of Hypertension (ISH). J Hypertension. 2023,41:000–000, https://doi.org/10.1097/HJH.0000000000003480
5. Whelton PK, Carey RM, Aronow WS, Casey DE, Collins KJ, Himmelfarb CD, et al. 2017 ACC/AHA/AAPA/ABC/ACPM/AGS/APhA/ASH/ASPC/NMA/PCNA Guideline for the prevention, detection, evaluation, and management of high blood pressure in adults: a report of the American

College of Cardiology/American Heart Association Task Force on clinical practice guidelines. J Am Coll Cardiol. 2018;71:e127–248, https://doi.org/10.1016/j.jacc.2017.11.006.

6. ElSayed NA, Aleppo G, Aroda VR, Bannuru RR, Brown FM, Bruemmer D, et al.; American Diabetes Association. 2. Classification and Diagnosis of Diabetes: Standards of Care in Diabetes-2023. Diabetes Care. 2023;46(Suppl 1): S19-S40, https://doi.org/10.2337/dc23-S002.
7. Levey AS, Stevens LA, Schmid CH, Zhang YL, Castro AF 3rd, Feldman HI, et al., CKD-EPI (Chronic Kidney Disease Epidemiology Collaboration). A new equation to estimate glomerular filtration rate. Ann Intern Med. 2009;150:604–12, https://doi.org/10.7326/0003-4819-150-9-200905050-00006.
8. Kidney Disease: Improving Global Outcomes (KDIGO) CKD Work Group. KDIGO 2012 Clinical practice guideline for the evaluation and management of chronic kidney disease. Kidney Int Suppl. 2013;3:5–14, https://doi.org/10.1038/kisup.2012.77.
9. Piepoli MF, Hoes AW, Agewall S, Albus C, Brotons C, Catapano AL, et al. 2016 European Guidelines on cardiovascular disease prevention in clinical practice. The Sixth Joint Task Force of the European Society of Cardiology and Other Societies on Cardiovascular Disease Prevention in Clinical Practice (constituted by representatives of 10 societies and by invited experts). Developed with the special contribution of the European Association for Cardiovascular Prevention & Rehabilitation (EACPR). Eur Heart J. 2016;37:2315–81, https://doi.org//10.1093/eurheartj/ehw106.
10. Cooney MT, Selmer R, Lindman A, Tverdal A, Menotti A, Thomsen T, et al. Cardiovascular disease risk estimation in older persons: SCORE O.P. Eur J Prev Cardiol. 2016;23:1093–103, https://doi.org/10.1177/2047487315588390.
